# A TYPOLOGY OF CARE-RELATED CHALLENGES THAT EMERGE FROM DEMENTIA FAMILY CAREGIVERS’ PHYSICAL PAIN

**DOI:** 10.1093/geroni/igae098.2737

**Published:** 2024-12-31

**Authors:** Shelbie Turner, Irina Mindlis, M Carrington Reid, Karl Pillemer

**Affiliations:** Weill Cornell Medicine, New York, New York, United States; Weill Cornell Medicine, New York, New York, United States; Weill Cornell Medicine, Weill Cornell Medicine, New York, United States; Cornell University, Ithaca, New York, United States

## Abstract

Many dementia family caregivers experience persistent physical pain. In addition to compromising quality of life, caregivers’ pain is associated with difficulty performing caregiving tasks and unmet needs in care recipients. This study developed a typology of care-related challenges that emerge as a consequence of pain among dementia family caregivers. We conducted semi-structured, in-depth interviews with 25 dementia family caregivers living with persistent pain. We inductively analyzed data via a thematic analysis approach to identify and categorize caregiving challenges stemming from caregivers’ pain. We then analyzed how challenges were related to one another and their consequences for caregiving outcomes. Analysis revealed three interrelated categories of caregiving challenges: 1) physical (e.g., inability to lift person with dementia), 2) psychological/emotional (e.g., worry about future progression of care), and 3) family/relational (e.g., resentment towards healthy family members for not helping with care). Caregivers reported that the co-occurrence of challenges compounded one another in ways that made both caregiving and pain management more difficult. For example, care recipients frequently became agitated when pain interfered with caregivers’ ability to perform care tasks (a family/relational challenge), which heightened caregivers’ negative emotions (a psychological/emotional challenge). Whether caregivers reported challenges and whether each challenge was connected to caregiving outcomes was dependent on various contextual factors, such as whether caregivers reported benefits from their own pain (e.g., greater empathy). The resulting typology offers a theoretical framework to guide future translational research on the topic of caregivers’ pain, including illuminating promising intervention targets of pain self-management programs for dementia family caregivers.

